# The role of insurance providers in supporting treatment and management of hepatitis C patients

**DOI:** 10.1186/s12913-019-3869-8

**Published:** 2019-01-10

**Authors:** Masoud Behzadifar, Hasan Abolghasem Gorji, Aziz Rezapour, Meysam Behzadifar, Nicola Luigi Bragazzi

**Affiliations:** 1 Department of Health Services Management, School of Health Management and Information Sciences, Iran University of Medical Sciences, Tehran, Iran; 2 Health Management and Economics Research Center, Iran University of Medical Sciences, Tehran, Iran; 3 Social Determinants of Health Research Center, Lorestan University of Medical Sciences, Khorramabad, Iran; 4 School of Public Health, Department of Health Sciences (DISSAL), University of Genoa, Genoa, Italy

**Keywords:** Hepatitis C virus, Treatment, Insurance, Policy

## Abstract

Today, one of the most important global public health challenges is represented by hepatitis C virus (HCV), which imposes relevant costs. Globally speaking, the median cost of HCV-related complications ranges from $280 for an uncomplicated hepatitis to $139,070 for a liver transplantation. There are effective therapies for HCV patients worldwide, which has increased the hope of improving the process of managing and curing these patients. The adherence of patients to the pharmacological treatment and the use of effective drugs in the management of HCV disease are of crucial importance for health policy- and decision-makers. Studies show that, globally, insurance coverage for patients with HCV is not adequate in that still many patients are not covered by insurance programs. This issue as well as the economic conditions of countries are very serious challenges for ensuring an effective treatment. The most important and greatest help currently available to ensure HCV treatment is to implement plans to reduce costs and support patients. Some studies have shown that the expansion of coverage by private payers seems able to generate positive spillover benefits to public insures**.** Insurers, in addition to maintaining and increasing their own interests, are trying to increase their social status as a sponsor of patients. In conclusion, HCV disease requires serious policies and affordable insurance coverage.

## Main text

### HCV epidemiology

Today, one of the most important global public health challenges is represented by hepatitis C virus (HCV), which imposes a dramatically relevant burden in terms of morbidity and mortality in many parts of the world. As such, the health sectors of the different countries have designed and implemented various programs for preventing, controlling and treating HCV [1]. According to the World Health Organization (WHO), 399,000 people die annually due to HCV and its complications, such as cirrhosis and liver cancer. This represents a major source of concern for health decision- and policy-makers [2].

There are effective therapies for HCV patients worldwide, which has increased the hope of improving the process of managing and curing these patients [3]. With the emergence of new viral treatments such as direct-acting antivirals (DAAs), the health sectors of the different countries are working to provide treatment for these people in order to increase their quality of life (QOL) and prevent complications related to the disease [4].

### HCV-generated economic burden

HCV is responsible for a huge economic burden for the countries of the world [5]. Direct costs (related to medical expenses for hepatic and extra-hepatic complications of HCV) as well as indirect costs (incurring from impaired QOL, and the loss of work productivity due to inability) have become a real challenge worldwide [6, 7]. In particular [8], according to a recent systematic review and meta-analysis of 102 studies, extra-hepatic complications such as diabetes (occurring in 15% of patients) and depression (occurring in approximately 25% of patients) are the major drivers of such relevant economic burden. Due to financial constraints, the health systems of many countries, in many cases, are not able to cover most of the costs generated by the treatment for these patients [7]. Direct, indirect, cumulative and lifetime costs generated by HCV vary according to the setting and the health system adopted; estimates computed for different countries are reported in Table 1 [9–19].

**Table 1:** Direct, indirect, cumulative and lifetime costs generated by hepatitis C virus (HCV) infection

Country/Reference	Direct costs	Indirect costs	Cumulative cost	Lifetime cost
USA [9]			$6.5 ($4.3–8.4) billion, increasing to $9.1 ($6.4–13.3) billion in 2024	$64,490
Canada [10]	56–81% (due to cirrhosis and its complications)	19–44%		$64,694
Germany [11]	Mean total cost *per* patient for hepatic complications €556–1425, for extra-hepatic complications €1921-3547, for chronic HCV €116–577, for non-chronic HCV-related pharmacy costs €1479–3719		Mean total cost *per* patient €5430-10,108	
Belgium [12]	€126 million (€30–257 million)		€1850 million	
France [13]	84% (47% due to liver cirrhosis, 18% to hepatocarcinoma and 19% to liver transplantation)	16%	€65,956,938	
Spain [14]	71.5%	28.5%	Mean total cost *per* patient €3198	
UK [15]				£97,555–125,359 for disability, £26,424–32,235 for healthcare
Ireland [16]			Mean total cost *per* patient €38,286–62,457 (interferon-based regimen), €55,734–81,873 (interferon-free regimen)	
Italy [17]	39.4%	60.6%	€1.06 billion	
Iran [18]	Total annual cost *per* patient USD1625.50, USD6,117.2, and USD11,047.2 for chronic HCV, cirrhosis, and hepatocellular carcinoma			
Worldwide [19]	Median cost of liver transplantation $139,070 ($15,430-443,700), refractory ascites $16,740 ($8990-35,940), hepatocellular carcinoma $15,310 ($3370-84,710), decompensated cirrhosis $14,660 ($3810-48,360), variceal hemorrhage $12,190 ($3550-46,120), hepatic encephalopathy $9180 ($5370-50,120), diuretic sensitive ascites $3400 ($1320-7470), compensated cirrhosis $820 ($50–2890), and chronic hepatitis C $280 ($90–1860).			

### The role of insurance programs

Economic-financial crises, rising HCV infection incidence rates, the availability of new biomedical technologies, and increased staff costs are the main factors that have limited the allocation of funding for the health sector and, as such, healthcare decision- and policy-makers are not able to meet with all the population’s health needs to properly satisfy them [20].

As a result, many patients face serious problems in having their disease properly managed and treated. This has prevented the establishment of a universal financial protection for these patients from the health sectors. In the meantime, increased out-of- pocket (OOP) expenditure has made it difficult for the poor people to meet their health-related needs [21].

The adherence of patients to the pharmacological treatment and the use of effective drugs in the management of HCV disease are of crucial importance for health decision- and policy-makers. Despite the effectiveness of DAAs [22], drug prices are high, and many patients are not able to pay for these costs. Therefore, many of them cannot use these medications and the treatment process is facing serious challenges [23].

The WHO reported in 2013 on trends in countries’ policies and programs related to hepatitis. In this report, many countries have referred to drugs for the treatment of HCV patients for activities such as drug subsidies, but one of the things that should be considered by health decision- and policy-makers is the lack of insurance coverage for these patients. In developed and developing countries, insurers do not play, indeed, a major role in helping/assisting HCV patients [24].

Studies show that, globally, insurance coverage for patients with HCV is not adequate in that still many patients are not covered by insurance programs. This issue and the economic conditions of countries represent very serious challenges for ensuring an effective treatment [25, 26].

Insurers have a very important role to play in supporting the health sector in creating financial resources and helping people to offset the financial catastrophe by offering support. Insurance should improve the process of treating HCV disease and play a more vibrant role in facilitating this process [27], ensuring, for instance, the continuity between HCV screening and healthcare (the so-called HCV screening and linkage-to-care or SLTC *continuum* or care cascade, which include different subsequent steps, namely: diagnosis, linkage to care, retention in care, prescription of antiretroviral therapy, and sustained virologic response/viral suppression) [28].

Medical expenses are causing poverty and financial catastrophe to patients and their families [29], and the payout increases from the OOP. Unfortunately, in patients with HCV, the increase in OOP has made the treatment problematic [30]. On the other hand, those who have been insured have been able to enjoy good antiviral treatment and, in addition to recovery, this will reduce the HCV-generated economic burden [31].

### The need for a consolidation and integration of the various insurance programs

There are diverse insurance providers, either public or commercial, offering a variety of programs and plans, such as government public programs for employees, or for urban and rural areas/settings, and government-provided catastrophic health insurance, or commercial health insurance programs. As such, creating an alliance for insurance managers is very important, to meet with different health needs, specific populations and curb costs, while, at the same time, delivering high-quality healthcare services. Consolidation and integration of payers and providers, leading to a “mosaic of insurance programs” can result in greater value and clinical safety in a sustainable way. “In a typical integrated network, the payers stipulate a framework whereby provider groups agree to care for a specific patient population with the goal of reaching or surpassing a predetermined set of quality and cost benchmarks. The integration model encourages healthcare organizations to deliver value to patients and reduce the overuse of treatments” [32].

Insurers should understand the conditions of the disease and the treatment of patients. Many insurance companies are unwilling to participate in the treatment of HCV patients due to their high costs. Meanwhile, some healthcare decision- and policy-makers seem to have overlooked the importance of treating these patients and are indifferent to them [33].

Due to the economic conditions of countries and the crises that every day affect the health system, negotiating with insurance managers is becoming more and more fundamental. If we want to achieve the goal of eliminating/eradicating HCV by the year 2030 [34], in addition to the Ministry of Health (MoH), other organizations, such as insurance companies, should be the main trustees that provide healthcare services to patients.

Treatment and adherence to patients are not complete unless the cost of treatment for them is lower and affordable and treatment provides these patients with better conditions for control and prevention. Reducing this disease, especially by increasing the use of injectable drugs, can be very effective in improving the overall health of the population [25].

### Obstacles and barriers for insurers in ensuring treatment access to HCV patients

There are different obstacles and barriers to ensure treatment access for HCV patients. For instance, there is a gap between health decision- and policy-makers and insurance managers/providers, and this could be a serious warning for the treatment of HCV patients. This gap can be due to different goals, aims, and perspectives (public health and societal point of view versus commercial purposes).

In order to cope with the high costs of the new DAA regimens, some insurers are restricting access to medications, establishing selective criteria for reimbursement. Gowda and collaborators [35] performed a prospective cohort study among American HCV patients. Authors found that absolute denials of DAA regimens by insurers have remained high and increased over time.

It should be emphasized that the health department is not a closed system and it has to use the high and effective potential of insurance programs. Insurers offering support to target and beneficiary groups can play a very valuable role in the treatment process. Health decision- and policy-makers can provide their support for HCV treatment by effectively communicating with insurers [36].

### Opportunities for insurers

The most important and greatest help currently available to guarantee HCV treatment is to implement plans to reduce costs and support patients. Of course, it should not be forgotten that insurers have a benefit in addition to their support for the health plan, which should also be addressed by policy- and decision-makers. Governments can give them concessions to obtain insurance, and provide insurance companies with incentives to support patients for further economic-financial coverage [26]. In the USA, Medicaid reimbursement [37] for therapeutics needed for the proper management and treatment of HCV infection has increased from $723 million to $2.35 billion in the period 2012–2015. Significant variations in the Medicaid reimbursement scheme for DAAs between states in 2014 could be detected that became even more evident in 2015. Expansion states were characterized by a higher increase in reimbursement for DAAs *per* individual suffering from HCV infection with respect to non- or late-expansion states, adjusting for pre-expansion reimbursement. In conclusion, approximately a third of states contributed more than 5–15% of pharmacy reimbursements to DAAs. New healthcare policies are, therefore, urgently needed in order to ensure coverage for an increasing number of expensive drugs required for the effective treatment of a rising number of patients.

Furthermore, according to a mathematical model analysis [38], the pricing scheme of drugs for the management of HCV infection seems to follow a value-based model, with a rather steady ratio of costs *per* achieved sustained virologic response over a period of 25 years. This leads to the paradoxical *conundrum* that healthcare systems are challenged by the economic-financial issues arising by the high resource utilization of these new drugs despite their cost-effectiveness [39] and their short- and long-term benefits in terms of health. Therefore, a synergy among the different stakeholders (pharmaceutical industries, healthcare payers and insurance providers) should be sought and established in order to find innovative drug pricing schemes to manage all the HCV patients.

Treating all eligible Medicaid patients [40], regardless of fibrotic stage, was computed to result in significantly fewer liver transplants and cases of cirrhosis, hepatocellular carcinoma and HCV-related deaths, as well as in significantly additional life-years and quality-adjusted life-years *per* patient. Treating all Medicaid chronic HCV patients was projected to result in significant cost savings and total costs of care reduction. However, the “treat-all” strategy appears to be promising and cost-effective, if properly supported by new funding and payment schemes, such as cost-sharing mechanisms [41], like plan premiums, deductibles, co-payments, and coinsurance, among others [42].

### The roles of the different insurance programs

The role of health insurance programs is recognized as a major contribution to the health sector and should be used to promote and improve health plans. Insurers can ensure pharmacological treatment and management to many poor people unable to receive health services, and governments can diversify their healthcare service packages. Insurers, in addition to maintaining and increasing their own interests, are trying to increase their social status as a sponsor of patients.

However, discrepancies between public and private/commercial insurance providers do exist. Such discrepancies could be due to economic factors. For instance, Mukka et al. [26], in a retrospective study of 160 HCV patients, found that patients with private insurance were more likely to receive treatment compared to patients with public aid. Vu and colleagues [43] performed a multivariate regression analysis and found that predictors of fewer steps in the authorization cascade were having non-Medicaid insurance, and being HCV non genotype 2. At the survival analysis, non-Medicaid insurance and mid-range fibrosis were significantly associated with fewer days to the authorization approval.

On the other hand, the role of private/commercial insurance providers can be positive. Moreno and collaborators [44] have modeled the costs and spillover effects of private insurers’ coverage and have found that, with expanded HCV treatment coverage, private payers may reduce medical expenditures, and, over a 20-year period, may experience even overall savings, also generating positive spillover effects to insures like Medicare.

## Conclusions

In conclusion, HCV disease is a challenging public health issue, that requires serious policies and affordable insurance coverage. Patients need to receive drugs, and bad economic conditions can represent barriers to the treatment, leading to poor access to healthcare. Negotiations between health decision- and policy-makers and insurance managers, either public or private, should be taken seriously. Preventing a higher incidence of disease should be a major concern for them. With the increasing incidence of disease in recent years, support for ensuring treatment of patients is very important and everyone should feel the onus to try to reduce the disease. Financial support for patients with HCV is very important and patients should not be liable for their costs. Insurance is an effective leverage for the adherence of all patients to treatment.

## Abbreviations

DAAs: Direct-acting antivirals
HCV: Hepatitis C virus
MOH: Ministry of Health
OOP: Out-of- pocket
QOL: Quality of life
SLTC: Screening and linkage-to-care
WHO: World Health Organization

## References

[1] Stanaway JD, Flaxman AD, Naghavi M, Fitzmaurice C, Vos T, Abubakar I (2016). The global burden of viral hepatitis from 1990 to 2013: findings from the global burden of disease study 2013. Lancet.

[2] World Health Organization. Hepatitis C, Fact sheet 2017 [Available from: http://www.who.int/mediacentre/factsheets/fs164/en/ (Access 16 February 2018).

[3] Umar M, Akhter TS (2016). New direct acting antiviral agents for the treatment of hepatitis C: 2016 and beyond. J Coll Physicians Surg Pak.

[4] Lynch SM, Wu GY (2016). Hepatitis C virus: a review of treatment guidelines, cost-effectiveness, and access to therapy. J Clin Transl Hepatol.

[5] Wong JB (2006). Hepatitis C: cost of illness and considerations for the economic evaluation of antiviral therapies. PharmacoEconomics.

[6] Jhaveri R, Grant W, Kauf TL, McHutchison J (2006). The burden of hepatitis C virus infection in children: estimated direct medical costs over a 10-year period. J Pediatr.

[7] Stepanova M, Younossi ZM (2017). Economic burden of hepatitis C infection. Clin Liver Dis.

[8] Younossi Z, Park H, Henry L, Adeyemi A, Stepanova M (2016). Extrahepatic manifestations of hepatitis C: a meta-analysis of prevalence, quality of life, and economic burden. Gastroenterology.

[9] Razavi H, Elkhoury AC, Elbasha E, Estes C, Pasini K, Poynard T (2013). Chronic hepatitis C virus (HCV) disease burden and cost in the United States. Hepatology.

[10] Myers RP, Krajden M, Bilodeau M, Kaita K, Marotta P, Peltekian K (2014). Burden of disease and cost of chronic hepatitis C infection in Canada. Can J Gastroenterol Hepatol.

[11] Kraus MR, Kleine H, Thönnes S, Pignot M, Sanchez GY (2018). Clinical and economic burden of hepatic and extrahepatic complications from chronic hepatitis C: a retrospective analysis of german sickness fund data. Infect Dis Ther.

[12] Vandijck D, Moreno C, Stärkel P, Van Damme P, Van Vlierberghe H, Hindman SJ (2014). Current and future health and economic impact of hepatitis C in Belgium. Acta Gastroenterol Belg.

[13] Rotily M, Vainchtock A, Jouaneton B, Wartelle-Bladou C, Abergel A (2013). How did chronic hepatitis C impact costs related to hospital health care in France in 2009?. Clin Res Hepatol Gastroenterol.

[14] Sicras-Mainar A, Navarro-Artieda R, Sáez-Zafra M (2018). Comorbidity, concomitant medication, use of resources and healthcare costs associated with chronic hepatitis C virus carriers in Spain. Gastroenterol Hepatol.

[15] Connolly MP, Kotsopoulos N, Ustianowski A (2018). Modeling the fiscal costs and benefits of alternative treatment strategies in the United Kingdom for chronic hepatitis C. J Med Econ.

[16] Gray E, O'Leary A, Kieran JA, Fogarty E, Dowling T, Norris S (2016). Direct costs of interferon-based and interferon-free direct-acting antiviral regimens for the treatment of chronic hepatitis C infection. J Viral Hepat.

[17] Marcellusi A, Viti R, Capone A, Mennini FS (2015). The economic burden of HCV-induced diseases in Italy. A probabilistic cost of illness model. Eur Rev Med Pharmacol Sci.

[18] Zare F, Fattahi MR, Sepehrimanesh M, Safarpour AR (2016). Economic burden of hepatitis C virus infection in different stages of disease: a report from southern Iran. Hepat Mon.

[19] El Khoury AC, Wallace C, Klimack WK, Razavi H (2012). Economic burden of hepatitis C-associated diseases: Europe, Asia Pacific, and the Americas. J Med Econ.

[20] Olsen IT (1998). Sustainability of health care: a framework for analysis. Health Policy Plan.

[21] Riggs KR, Ubel PA (2014). Overcoming barriers to discussing out-of-pocket costs with patients. JAMA Intern Med.

[22] He T, Lopez-Olivo MA, Hur C, Chhatwal J (2017). Systematic review: cost-effectiveness of direct-acting antivirals for treatment of hepatitis C genotypes 2-6. Aliment Pharmacol Ther.

[23] Rosenthal ES, Graham CS (2016). Price and affordability of direct-acting antiviral regimens for hepatitis C virus in the United States. Infect Agent Cancer.

[24] World Health Organization. Global policy report on the prevention and control of viral hepatitis in WHO Member States 2013 2013 [Available from: http://www.who.int/hiv/pub/hepatitis/global_report/en/ (Access 13 February 2018).

[25] Stepanova M, Younossi ZM (2015). Interferon-free regimens for chronic hepatitis C: barriers due to treatment candidacy and insurance coverage. Dig Dis Sci.

[26] Mukka M, Akram S, Koirala J (2017). Impact of insurance and treatment regimens on HCV outcome: long-term follow-up study. Open Forum Infect Dis.

[27] Habib SS, Perveen S, Khuwaja HMA (2016). The role of micro health insurance in providing financial risk protection in developing countries- a systematic review. BMC Public Health.

[28] Mulligan K, Sullivan J, Yoon L, Chou J, Van Nuys K (2018). Evaluating HCV screening, linkage to care, and treatment across insurers. Am J Manag Care.

[29] Pauly MV, Zweifel P, Scheffler RM, Preker AS, Bassett M (2006). Private health insurance in developing countries. Health Aff.

[30] Jung JK, Feldman R, Cheong C, Du P, Leslie D. Coverage for hepatitis C drugs in medicare part D. Am J Manag Care. 2016 22(6 Spec No):SP220–6. PMC5738242 27266952

[31] Federico CA, Hsu PC, Krajden M, Yoshida EM, Bremner KE, Weiss AA (2012). Patient time costs and out-of-pocket costs in hepatitis C. Liver Int.

[32] Vogenberg FR, Santilli J (2018). Healthcare trends for 2018. Am Health Drug Benefits.

[33] Ward JW, Valdiserri RO, Koh HK (2012). Hepatitis C virus prevention, care, and treatment: from policy to practice. Clin Infect Dis.

[34] Hellard M, Sacks-Davis R, Doyle J. Hepatitis C elimination by 2030 through treatment and prevention: think global, act in local networks. J Epidemiol Community Health. 2016;24:jech-2015-205454. 10.1136/jech-2015-205454 27343304

[35] Gowda C, Lott S, Grigorian M, Carbonari DM, Saine ME, Trooskin S, et al. Absolute insurer denial of direct-acting antiviral therapy for hepatitis C: a national specialty pharmacy cohort study. Open Forum Infect Dis. 2018;5(6):ofy076. 10.1093/ofid/ofy076 PMC6016397 29977955

[36] Levy H, Meltzer D (2008). The impact of health insurance on health. Annu Rev Public Health.

[37] Lu CY, Zhang F, Golonski N, Lupton C, Jeffrey P, Wagner AK (2018). State medicaid reimbursement for medications for chronic hepatitis C infection from 2012 through 2015. Value Health.

[38] Vernaz N, Girardin F, Goossens N, Brügger U, Riguzzi M, Perrier A (2016). Drug pricing evolution in hepatitis C. PLoS One.

[39] Coward S, Leggett L, Kaplan GG, Clement F (2016). Cost-effectiveness of screening for hepatitis C virus: a systematic review of economic evaluations. BMJ Open.

[40] Younossi Z, Gordon SC, Ahmed A, Dieterich D, Saab S, Beckerman R (2017). Treating medicaid patients with hepatitis C: clinical and economic impact. Am J Manag Care.

[41] Lakdawalla DN, Linthicum MT, Vanderpuye-Orgle J. Does patient cost sharing for HCV drugs make sense? Am J Manag Care. 2016;22(6 Spec No):SP188–90. 27266947

[42] Rodriguez C, Reynolds A (2016). Accessing the cure: helping patients with hepatitis C overcome barriers to care. Am J Manag Care.

[43] Vu TM, Toribio W, Riazi F, Ciprian G, Gibbs N, Giardina M (2018). Increasing access to hepatitis C virus medications: a program model using patient navigators and specialty pharmacy to obtain prior authorization approval. J Manag Care Spec Pharm.

[44] Moreno GA, Mulligan K, Huber C, Linthicum MT, Dreyfus D, Juday T, et al. Costs and spillover effects of private insurers' coverage of hepatitis C treatment. Am J Manag Care. 2016;22(6 Spec No):SP236–44. 27266954

